# A comprehensive study on the effect of carbonization temperature on the physical and chemical properties of carbon fibers

**DOI:** 10.1038/s41598-022-15085-x

**Published:** 2022-06-23

**Authors:** Roya Shokrani Havigh, Hossein Mahmoudi Chenari

**Affiliations:** Department of Physics, Faculty of Science, University of Guilan, Namjoo Ave, Po Box 41335-1914, Rasht, Iran

**Keywords:** Materials science, Nanoscience and technology, Physics

## Abstract

Carbon fibers were successfully fabricated via the electrospinning technique, followed by stabilizing and carbonizing electrospun PAN fibers. A wide range of analytical techniques such as scanning electron microscopy (SEM), transmission electron microscopy (TEM), X-ray diffraction (XRD), Diffuse reflectance spectroscopy (DRS), photoluminescence spectroscopy (PL), vibrating sample magnetometer (VSM) techniques, and Hall effect were performed to study of the effect of carbonization temperature on the physical and chemical characterization of carbon fibers. The SEM images of the PAN precursor exhibit a smooth outer surface, after the stabilization and carbonization process, along with a broken fiber at higher carbonization temperature about 1400 °C. Morphological characterization based on the recorded TEM images of carbonized fibers at 1000 °C and 1400 °C, showed that the obtained morphology can be classified as fiber structures, where their diameters ranged from 196 to 331 nm. The XRD patterns of PAN-based carbon fibers confirm the structural changes from linear structure into a graphite-like structure. The DRS study indicates the possible π–π*/σ–π* and n–π* transitions. The presence of the surface functional groups and different trapped radiative recombination on the emission bands is confirmed by the PL. VSM results shows the weak ferromagnetic nature of the carbon fibers.

## Introduction

Carbon is one of the most amazing elements in nature with a wide range of structure and properties. The crystalline allotropic forms of carbon^1^ with a regular geometrical shape are Graphite, diamond, and fullerene, whereas carbon nanofiber, carbon nanotube, and carbon black can be regarded as turbostratic allotropic forms of carbon^2^. Turbostratic structure is between the amorphous carbon phase and crystalline graphite phase. Graphite phase contains sp^2^ hybridized carbon atoms, whereas amorphous carbon atoms is derived from the varying amounts of sp^3^ hybridized carbon atoms along with a small amount of sp^2^-bonded atoms. Turbostratic carbon also has a graphite-like layered structure that the layers are bent^3^, and do not show stacking sequence.

Among the carbon allotropes, carbon nanofibers as sp^2^-based discontinuous linear filaments have attracted much attention in recent years^4^. Carbon fibers possess high mechanical strengths and modules, strong fatigue and corrosion resistance, high stiffness, low weight, and excellent electrical and thermal conductivities^5–7^. Therefore, they have been widely used for numerous applications like rechargeable battery electrode materials in electrochemical capacitor cells, electrochemical catalysis, hydrogen storage, and polymer reinforcement^8–13^.

Electrospun nanofibers exhibit noticeably different properties, such as nanosized diameter, high surface area, and tunable porosity, which make them applicable for the production of high-performance nanocomposites, tissue scaffolds, drug delivery systems, electrode materials, and energy storage devices^14,15^.

Among the nanofibers materials, carbon nanofibers, fabricated by pyrolyzing the spun fibers from an organic precursor (e.g. polyacrylonitrile (PAN)^16–19^, Polyvinylpyrrolidone (PVP)^20^, Polyvinyl alcohol (PVA)^21^, Polyamide^22,23^ and Pitch^24^), have been received much attention. PAN is the most common precursor for high-performance carbon nanofibers due to its high carbon yields and excellent mechanical properties.

PAN-based carbon fibers are produced through a three major steps as electrospinning, stabilization, and carbonization. The electrospinning technique has been considered as one of the advanced fiber fabrication techniques from the polymer solutions^23,25^. Stabilization is an essential step in the conversion of PAN fibers to high-performance carbon fibers. In stabilization, precursor fibers are heated to a temperature in the range of 200–300 °C for over an hour in the air. Stabilization alters the chemical structure of the fibers and causes them to become thermally stable^26^. During this process, the PAN structure undergoes cyclization, dehydrogenation, aromatization, oxidation, and crosslinking reactions that the triple bond ($\mathrm{C}{\equiv}\mathrm{N}$) converts to a double bond (C=N). As a result, the linear PAN chains convert into cyclic or ladder-like structures, which display the thermally stable polymer and prevent melting during the carbonization process^27,28^. Carbonization of the stabilized PAN fibers is carried out at temperatures ranging from 800 to 1500 °C in an inert atmosphere (nitrogen (N_2_) or Ar gases)^29^. Carbonization is done to remove non-carbon elements in the form of different gases. During this process, the fiber diameters are reduced and the fibers lose approximately 50% of their weight. Carbonization treatment under N_2_ increases the tensile and modulus of carbon fiber significantly^30,31^.

In order to improve the carbon fiber performance, the carbonized fiber must undergo a graphitization process. In graphitization, carbon fibers are heated at temperatures higher than 2000 °C which causes the growth of the ordered structure with high crystalline orientation and low interlayer spacing. Unlike the carbonization process, nitrogen cannot be used in the graphitization process, due to its reaction with carbon and the formation of cyanogen^32^.

The physical and chemical characterization of PAN-based carbon fibers are deeply influenced by the heat-treatment temperature. Hence, the final heat-treatment temperature plays an important role for having carbon fibers with improved properties. Also, at the elevated temperature, aromatic molecules are aligned more uniformly, and the carbon fibers is well ordered. As a result, some spatial properties of carbon fibers such as the strength, modulus, electrical and thermal conductivities increases with increasing the carbonization temperature^33,34^.

Recently, there have been some reports about the effect of carbonization temperature on the properties of Carbon fibers. Zhou et al.^17^ investigated the effect of carbonization temperatures on structural, mechanical, and electrical properties of carbon nanofiber. Their study revealed more graphitic and structurally ordered of the carbon nanofibers, along with an improved electrical conductivities and mechanical properties, at higher carbonization temperature. In another study, Arshad et al.^35^ synthesized carbon fibers from the optimum stabilization and carbonization temperatures between 800 and 1700 °C, leading to Turbostratic carbon crystallites with an improved elastic modulus and the tensile strength.

To the best of our knowledge, carbon fibers have not been investigated in detail in view of their structural, optical, magnetic, and electrical properties. Although the structure evolution of PAN-based carbon fibers has been reported in some literatures, but, there are lack of information about the detail characterization in view of the structure analysis using accurate X-ray peak profile by a curve fitting procedure and also a correlation between their related optical, magnetical and electrical properties. For the reason, in this paper, carbon fibers were prepared by stabilizing and carbonizing electrospun PAN fibers. Then, the effect of carbonization temperature on the morphology, structural, optical, magnetical, and electrical properties of the synthesized PAN-based carbon fibers was investigated by a wide range of analytical techniques such as scanning electron microscopy (SEM), X-ray diffraction (XRD), Diffuse reflectance spectroscopy (DRS), photoluminescence spectroscopy (PL), Fourier transform infrared spectroscopy (FT-IR), vibrating sample magnetometer (VSM) techniques, and Hall effect measurement. The obtained results showed good agreement between the chemical and physical characterization of the PAN-based carbon fibers.

## Experimental

### Materials

Polyacrylonitrile (PAN) (M_w_ = 150,000) was obtained from Sigma-Aldrich. The 99% N, N-Dimethylformamide (DMF) was also purchased from Sigma-Aldrich and used without further purification. Carbon fibers were prepared by Electroris (eSpinner NF CO-N/VI, Iran, http://www.anstco.com) with a high voltage 1–35 kV, followed by stabilization and carbonization using muffle furnace (FM8P, Iran, farazmaco.com) and tube furnace (TF5/25-1500, Iran,azarfurnace.com) respectively.

### Electrospinning of carbon fibers

To prepare the electrospinning solution, 0.5 g of PAN was dissolved in 10 mL of DMF and the solution was stirred continuously using the magnetic stirrer (Delta model HM-101) at room temperature for 24 h. The as-prepared solution was transferred into a plastic syringe with a 22-gauge metal needle. The solution was then electrospun at applied high voltage (15–25 kV) and needle to collector distance (15–20 cm) to obtain the optimal condition for the electrospun. The optimal condition was as follows: applied voltage 24 kV, needle to collector distance 18 cm, and flow rate 0.5 ml/h. After electrospinning, the fiber mat was collected from the aluminum foil (rolled on the rotating drum), stabilized, and carbonized.

### Stabilization and carbonization

**Figure 1:**
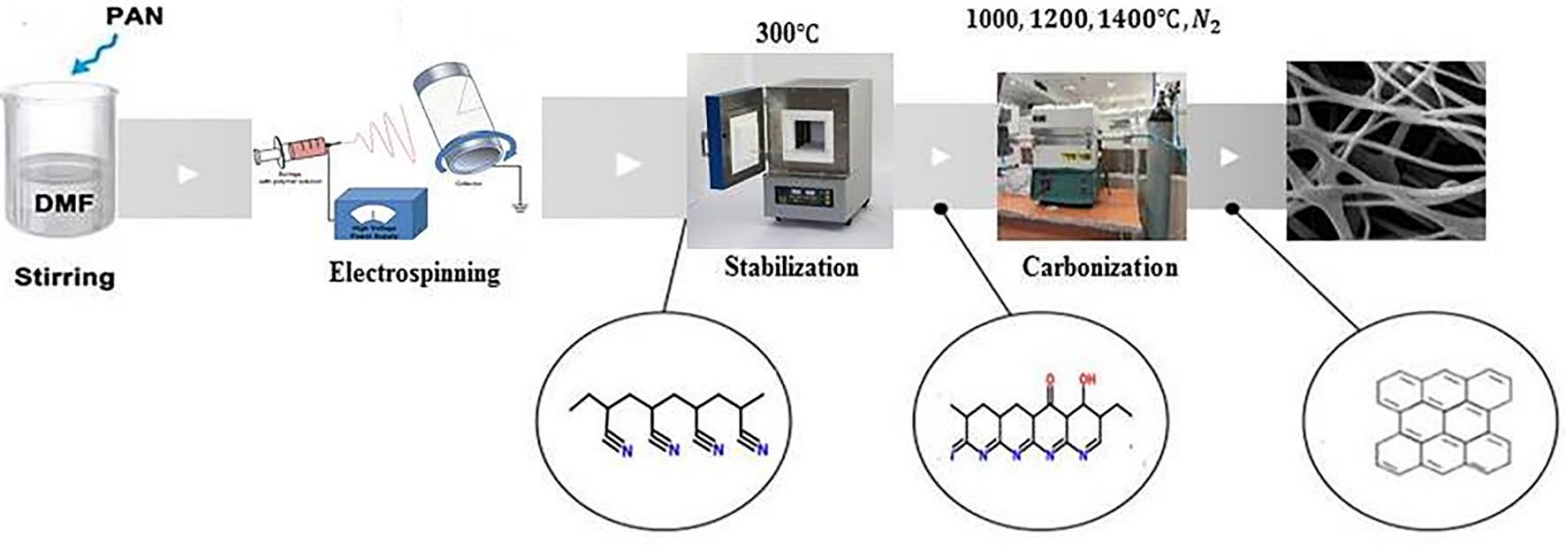
Schematic of the preparation process of carbon fibers.

As shown in the schematic of the preparation process of carbon fibers in the Fig. 1, stabilization step is one of the most important step that PAN-based fibers convert to the carbon fiber, as different chemical reactions occur and the structure of the carbon fiber is set to form the conjugated ladder structure. The stabilization was carried out by heating the PAN fibers at 300 °C with a heating rate of 5 °C/min for 1 h in air atmosphere. After the stabilization step, the fibers were carbonized in a high-temperature tube furnace at different temperatures of 1000, 1200, and 1400 °C for 1 h under a N_2_ gas flow (high purity nitrogen gas) with a heating rate of 10 °C/min. Fibers carbonized at 1000, 1200, and 1400 °C were denoted as C1, C2, C3, respectively. The carbon fiber preparation process is shown schematically in Fig. 1.

### Characterization

Scanning electron microscope (Stereo Scan 360) was applied to study the morphologies of carbon fibers. The elemental composition of fibers was obtained from energy-dispersive X-ray Spectrometer (EDS) equipped with the FESEM TESCAN MIRA3. A CM-120 Transmission Electron Microscope (TEM) was employed to study the carbonaceous structures in the carbonized PAN fibers. The structural characterization of carbonized PAN fibers was carried out by analyzing X-ray diffraction (XRD) patterns, obtained using a Philips XʼPert, X-ray diffractometer using CuKα radiation (Wavelength = 1.54056 Å) at 40 kV and 30 mA. The chemical structures of the fibers were analyzed by Fourier-transform infrared spectroscopy (Varian-3600 FT-IR spectrometer) whit KBr crystal in the infrared region between 4000 and 400 cm^−1^. The magnetic properties of samples were investigated using a vibrating sample magnetometer (VSM), magnetic daghigh kavir: MDKB measurement. Optical absorption studies were carried out using a DRS spectrophotometer. The PerkinElmer (Model: LS-55, pulsed Xenon lamp) was used to measure the PL spectra. The electrical conductivities of the PAN-based carbon fibers were measured with an ECOPIA Hall Effect sensor. The tests were carried out in a constant temperature and humidity chamber with a temperature of 25 °C and a humidity of 50%.

## Result and discussion

**Figure 2:**
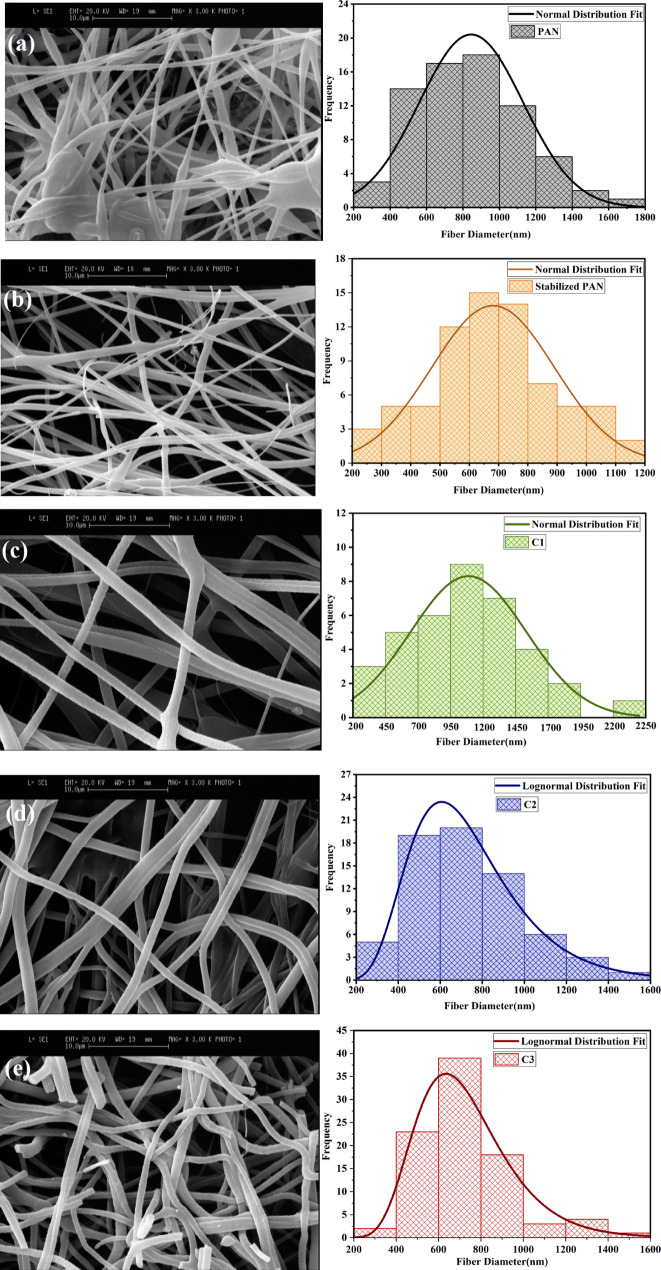
SEM micrographs and diameter size distribution of fibers, (**a**) PAN, (**b**) stabilized at 300 °C, (**c**) C1, (**d**) C2, and (**e**) C3.

**Figure 3:**
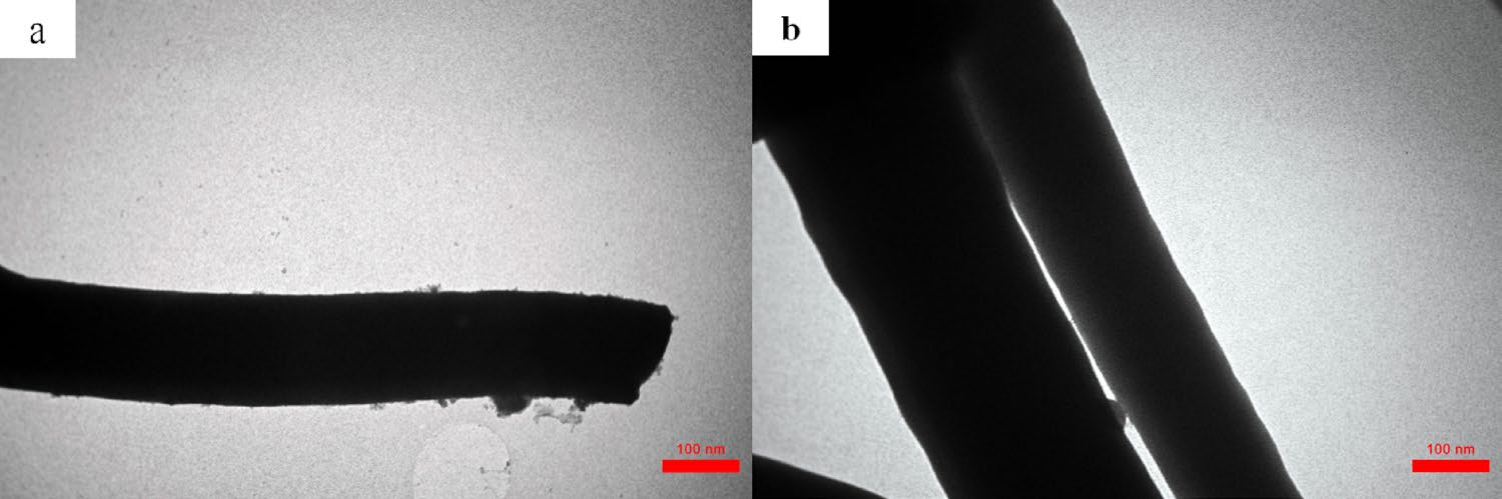
TEM images showing the representative microstructures of carbonized fibers at (**a**) 1000 °C, and (**b**) 1400 °C.

The morphology of the as-prepared PAN fibers, stabilized and carbonized fibers were investigated by SEM images, which was shown in the Fig. 2. Also, an image analyzing software (Digimizer) was used to get histogram of the diameter distributions of fibers. It’s evident from the Fig. 2a, that the PAN precursor fibers shows beaded fibers, separated in different directions, and oriented randomly owing to the bending instability of the spinning jet. It can be visualized that after stabilization and carbonization, some modifications occur in the fiber’s diameters distribution due to the elimination of unstable substances. Different amount of tension during the spinning can be one of the reasons for differences in fiber’s diameters and its distribution. According to the histogram of diameter distribution of the fibers, the estimated average fiber’s diameters decreased from 840 ± 34 to 718 ± 22 nm with increasing carbonization temperature to 1400 °C, due to the removal of volatile materials of low molar mass. After the stabilization and carbonization process, fibers exhibit a smooth outer surface (Fig. 2b–e). All fibers were continuous except for fibers prepared at carbonization temperature about 1400 °C. As the fiber network shows few fiber breakages along with a reduced length, due to the high-temperature activation that contributes to the removal of the volatile low molecular weight fractions. Such treatment confirms the temperature sensitivity of carbonized fibers. With increasing heat treatment, the thermal residual stress may affect the strength of fiber, resulting in fiber damage and eventually failure of the continues fiber morphology. Also, it is worth noting that different magnitude of thermal residual stresses, that are not homogeneously distributed in the fiber network, cause different degree of fiber fragmentation and fiber damage (here broken structure). Figure 3a,b show the TEM images of PAN fibers carbonized at 1000 °C and 1400 °C with a diameter of about 196 and 331 nm, respectively. Possibly the obtained fibers classified as one-dimensional. TEM images also confirms a smooth surface and the absence of any roughness for fibers.

**Figure 4:**
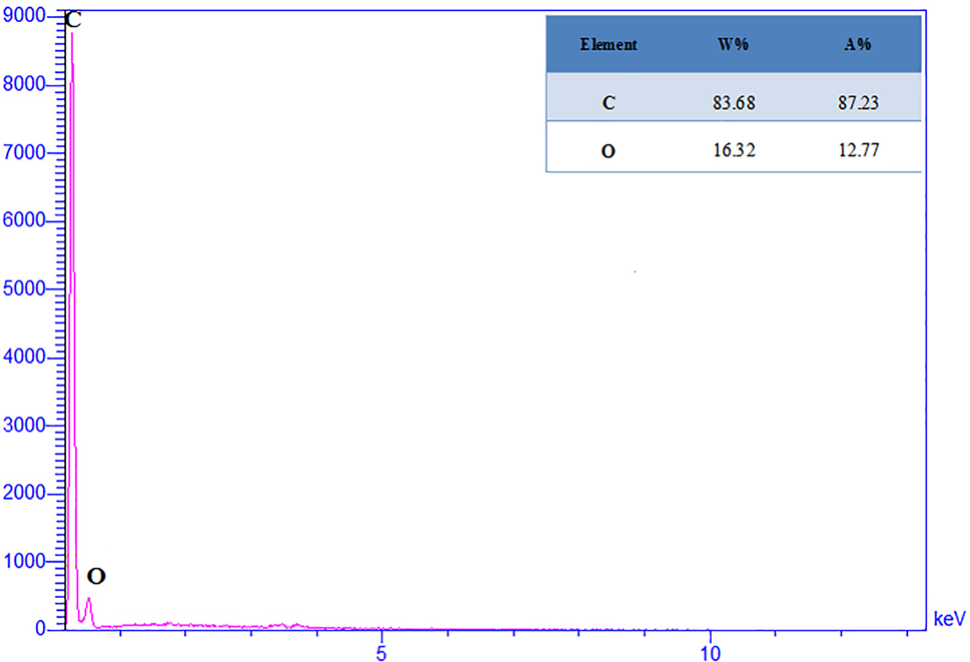
Energy dispersive X-ray spectrum of sample C2.

The elemental composition of the carbon fibers was studied by energy dispersive X-ray (EDX). EDX analysis was performed on sample C2 (Fig. 4). The observed peaks in 0.28 and 0.53 keV correspond to emission lines Ka1 of carbon and Ka1 of oxygen. The EDX analysis proves the successful preparation of carbon fibers.

**Figure 5:**
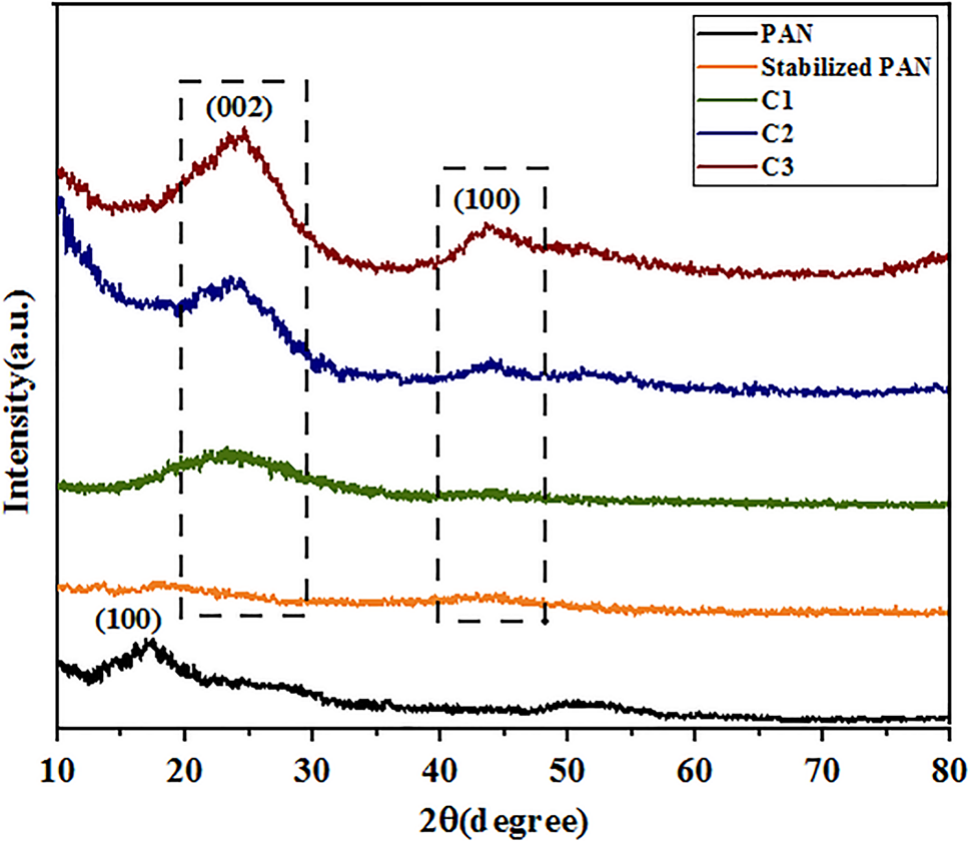
XRD patterns of as-electrospun, stabilized, and carbonized PAN fibers.

X-ray diffraction analysis (XRD) was applied to study the crystallographic characteristics of the carbon fibers. XRD shows obvious structural changes from linear structure into a graphite-like structure, that occur in PAN fibers as the removal of the non-carbon elements continues at carbonized stages. The XRD pattern of as-electrospun PAN fibers shows a diffraction peak at 2θ = 16.1°, corresponding to the (100) crystallographic plane in PAN^5^. This peak could be related to the hexagonal lattice, showing the strong intra-molecular dipole–dipole interactions of the $\mathrm{C}{\equiv} \mathrm{N}$ group in PAN fibers. After stabilization, the peak at 2θ = 16.1° disappeared, while a broad peak around the 2θ = 24° emerged; This was attributed to the weakness of the intermolecular action of nitrile groups, destruction of the original structure, and transformation of linear ordered structure into a ladder-like polymeric structures in the stabilized PAN^36^. As indicated in the Fig. 5, at this stage, diffractions are broad, implying formation of a very small and randomly arranged pseudo-graphite sheets.

The XRD patterns of carbonized PAN fibers show the diffraction peak at 2θ = 24°, attributed to the (002) crystallographic plane of graphite crystallites^37^. All carbonized fibers at different temperatures exhibit obvious (002) lattice diffraction peaks, indicating the formation of a layered graphite-type structure. With the increase of the final carbonization temperature, the intensity and sharpness of this peak gradually increased and the new peak appeared at 43°. This peak can be also assigned to the (100) graphite plane^38^. The main Bragg reflection (002) peak shows the evolution of a turbostratic graphitic structure with randomly oriented graphitic carbon layers, while the (100) peak is related to the carbon atoms on the same plane^39^. planar structures stack into layers for the fibers carbonized at 1400 °C, due to π–π interactions. It’s obvious that the intensity of the main peak (002) is much more prominent for the fibers carbonized at 1400 °C, indicating a higher degree of crystallinity. Scherrer’s equation was used to estimate the microstructure parameters of the PAN-based carbon fibers such as the inter planar spacing (d), the crystallite thickness (L_c_), and layer plane length (L_a_) from the x-ray peak profile analysis, neglecting the occurred strain. The d-spacing of the fibers was calculated by the Bragg equation as:

1 $$\mathrm{d}_{002}=\frac{\lambda }{2\mathrm{sin}\theta }$$

**Figure 6:**
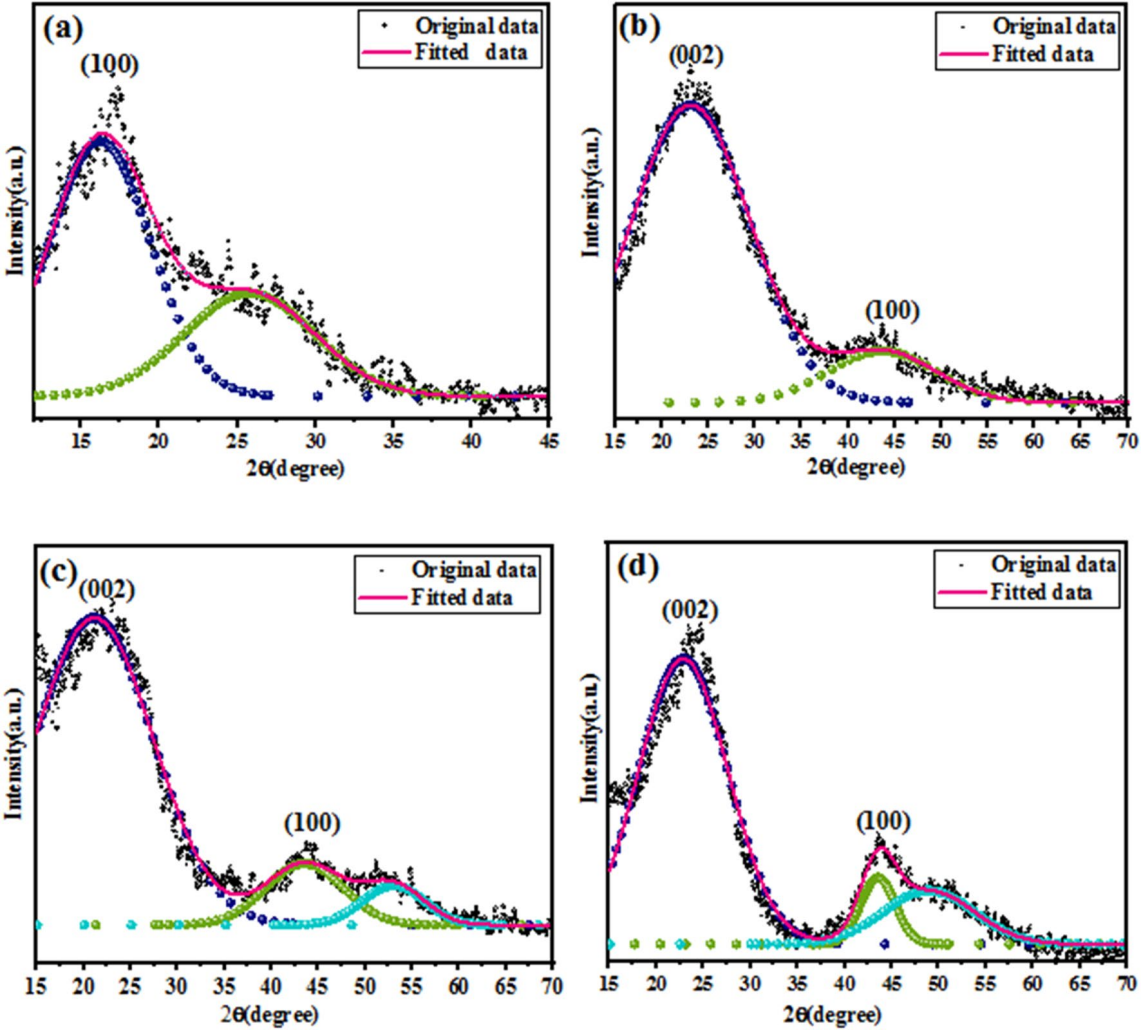
Deconvolution of XRD patterns of (**a**) as-spun PAN, (**b**) C1, (**c**) C2, and (**d**) C3 fibers.

The L_c_ and L_a_ were estimated by the Scherrer’s equation from the positions of the diffraction maxima and the width at half-maximum intensity of the (002) and (100) peaks^40^, as follows:

2 $${L}_{c}=\frac{k\lambda }{{\beta }_{002}{\mathrm{cos}\theta }_{002}} , { L}_{a}=\frac{k\lambda }{{\beta }_{10}{\mathrm{cos}\theta }_{10}}$$

where λ is the wavelength of X-ray used (0.154 nm), the form factor K is 0.91 for L_c_, and 1.84 for L_a_^41^, θ is the Bragg angle for the reflection concerned, and β is the full width at half maximum (FWHM) of X-ray diffraction intensity in radian. In order to obtain accurate peak parameters, a curve fitting procedure was utilized for X-ray diffraction (Fig. 6). The calculated values of d_002_, L_a_, and L_c_ are shown in Table 1. It was evident that the d_002_ value decreased, while the L_a_ and L_c_ values increased upon increasing the final carbonization temperature. Growth of lateral size, L_a,_ with increasing carbonized temperature can be due to the non-carbon atoms elimination reaction^41^. Consequently, the carbonization treatment favored the graphite crystallites to undergo structural rearrangements to become larger and well ordered.

**Table 1:** The average interplanar spacing ‘‘d”, the crystallite thickness ‘‘L_c_’’, and layer plane length ‘‘L_a_’’ of as-electrospun PAN and carbonized PAN fibers.

Sample	2θ_100_ (°)	L (nm)	d_100_ (nm)	2θ_002_ (°)	2θ_10_ (°)	β_002_ (radian)	Β_10_ (radian)	d_c_ (nm)	L_c_ (nm)	L_a_ (nm)
PAN	16.1	1.085	0.279	–	–	–	–	–	–	–
C1	–	–	–	23.2	43.6	0.26	0.237	0.195	0.57	1.65
C2	–	–	–	23.5	43.4	0.24	0.210	0.193	0.63	1.85
C3	–	–	–	24.1	43.8	0.19	0.195	0.188	0.78	2.00

**Figure 7:**
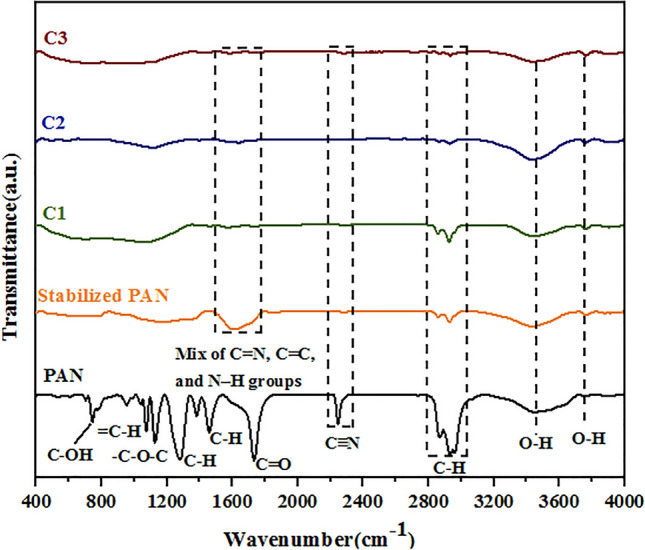
FTIR spectra of fiber samples in the range 400–4000 cm^−1^.

FTIR spectra of the as-electrospun, stabilized, and carbonized PAN fibers are shown in Fig. 7. In the as-electrospun fiber spectrum, the absorption peak at 2246 cm^−1^ is related to nitrile ($\mathrm{C}{\equiv}\mathrm{N}$) bonds. The peaks at 2868–2959 cm^−1^, 1459 cm^−1^, 1380 cm^−1^, and 1277 cm^−1^ are related to vibrations of the aliphatic CH groups (CH, CH_2_, and CH_3_)^42^. The peak corresponding to C=O stretching vibration is located at 1733 cm^−1^. After stabilization, the intensity of the peak associated with nitrile group at 2246 cm^−1^ decreased significantly and the intensity of the aliphatic CH groups reduced. The appearance of the peak at 1607 cm^−1^ is due to a mix of C=N, C=C, and N–H groups. The appearance of the C=C group is due to the dehydrogenation reaction. The most important structural change is the conversion of C$\equiv$N into C=N that results from cyclization and cross-linking reactions^34^. All the spectra display broadband at 3425 cm^−1^, corresponding to the O–H stretching vibration of chemisorbed water and a hydroxyl group. With increasing carbonization temperature, the peak intensity of CH_2_ at 2921 and 2851 cm^−1^ is reduced. The absence of these peaks indicates that the PAN was fully carbonized and hydrocarbon converted to the graphitic structure^43^.

**Figure 8:**
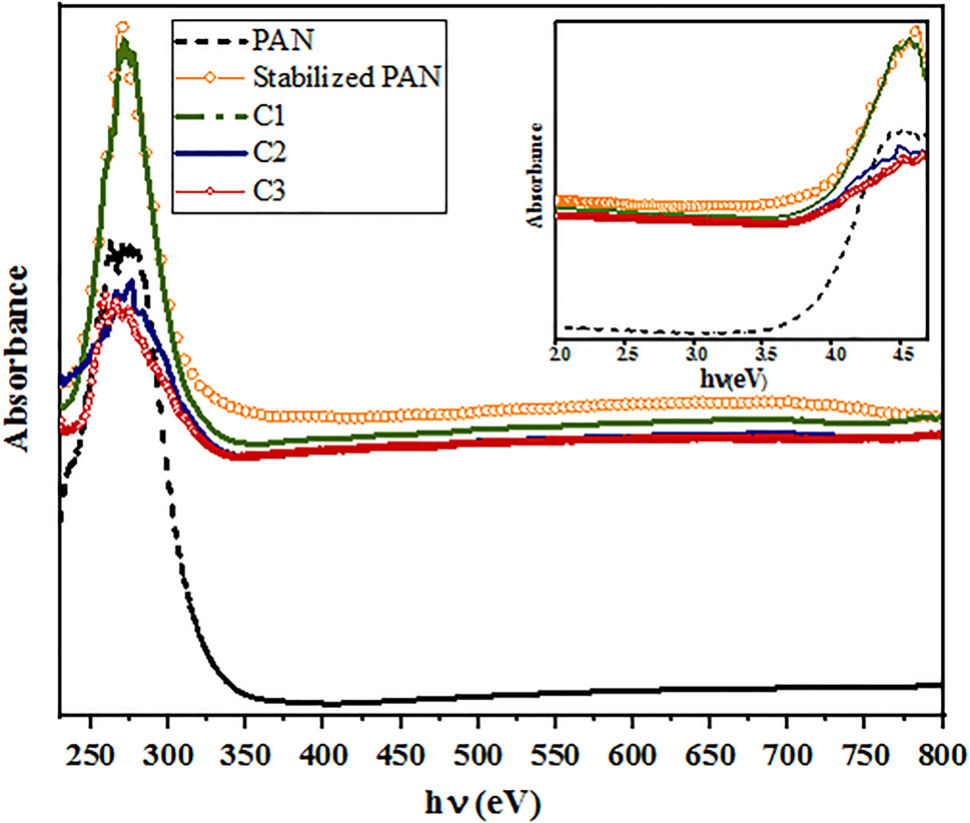
The absorbance spectra of PAN-based fibers. The inset shows the absorbance curve versus the photon energy (eV).

**Figure 9:**
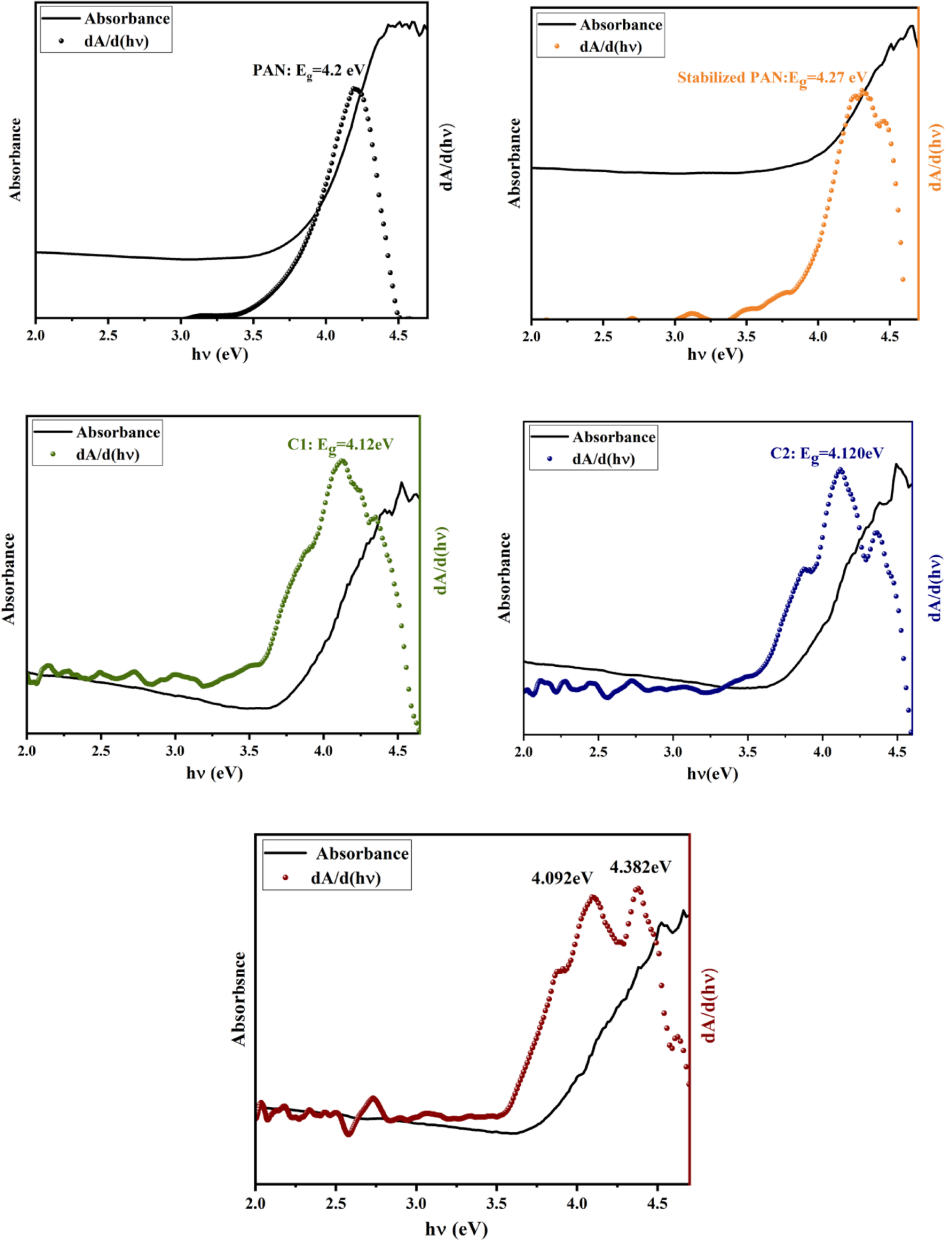
The first derivative of the absorbance data versus energy for PAN-based fibers.

Diffuse reflectance spectroscopy (DRS) and Photoluminescence spectroscopy (PL) were performed to study of optical properties of PAN-based carbon fibers. The DRS spectra (Fig. 8) show strong absorption around 272 nm. This absorption peak could be assigned to the $\pi \to {\pi }^{*}$ transition of the conjugated C=C band^44–46^, and the n–${\pi }^{*}$ transition of the C=O band^47^. Generation of conjugated plane with a relatively large conjugation length favored the increasing of the absorption peak. The optical band gap was estimated by the first derivative method of absorption data (Fig. 9). In this method, the optical band gap (E_g_) can be calculated from the maximum of the first derivative of the absorbance data with respect to the photon energy. The two sharp peaks show possible absorption mechanisms inside the fibers. The surface chemical groups may contribute to the absorption behavior of carbon fibers. The *π*–π*/*σ*–π* and n–π* transitions, related to the sp^2^-conjugated and surface-mediated in the groups on the carbon surface, respectively, are typical in the absorption spectra of carbon. It’s obvious that after structure changes of PAN-based fiber from linear into a graphite-like structure, the estimated band gap value decreased from 4.2 to 4.09 eV, in accordance with the related X-ray diffraction results, as the value of both the crystallite thickness (L_c_) and layer plane length (L_a_) increased. Also. It should be noted the related chemical bonds for the oxygen and nitrogen elements will induce different impurity levels in the band gap, leading to a change of optical transitions. The appearance another peak at E = 4.38 eV, could be assigned to the energetically most favorable σ(bonding) –π* (anti-bonding) transitions.

**Figure 10:**
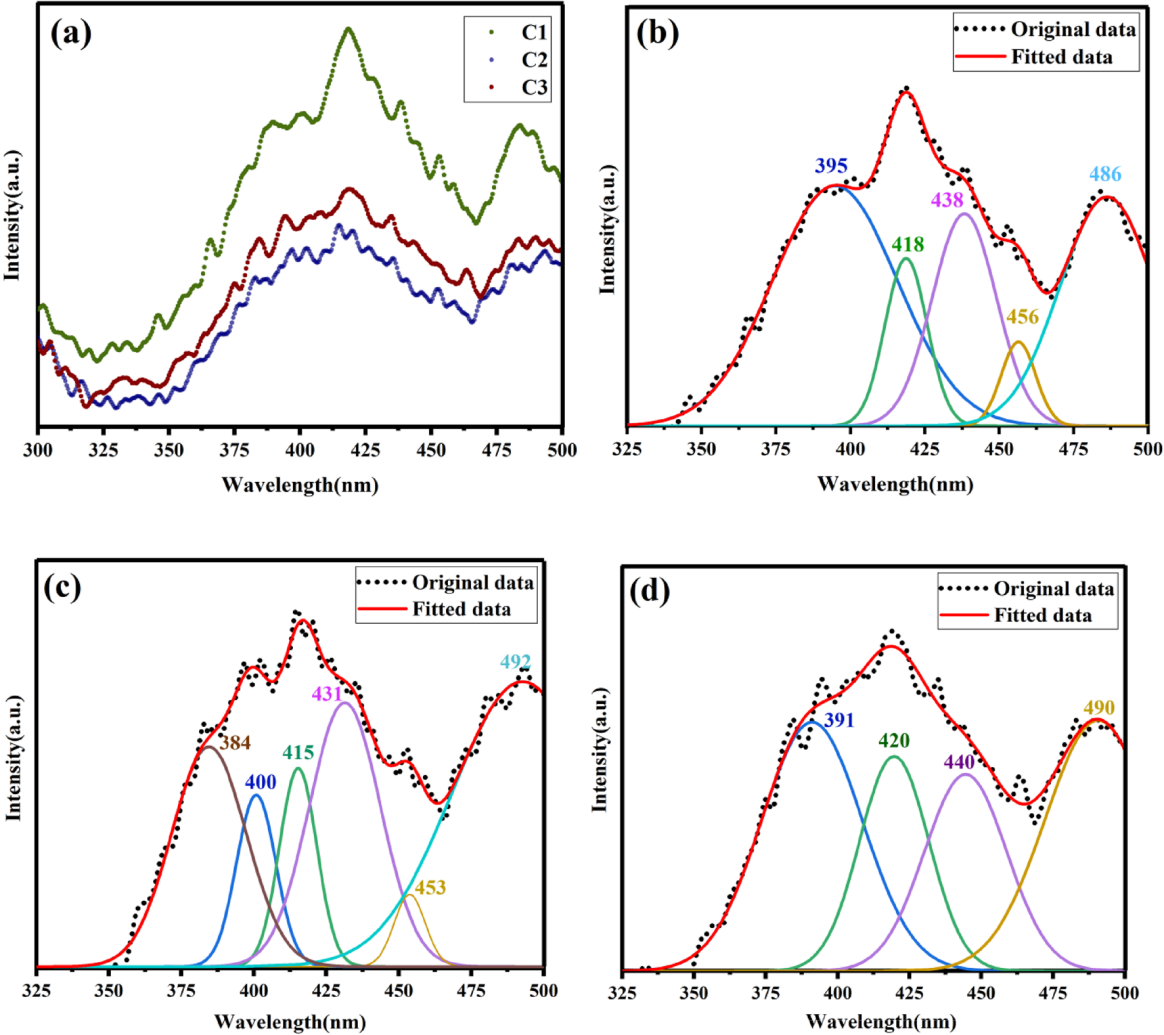
(**a**) The PL emission spectra of different samples. (**b**) PL spectrum deconvolution of the sample C1, (**c**) PL spectrum deconvolution of the sample C2, (**d**) PL spectrum deconvolution of the sampleC3.

Fluorescence spectroscopy (PL) was used to examine the excitonic energies and different recombination mechanisms of carbon fibers at excitation wavelengths of 270 nm. Figure 10 shows the PL spectra of fibers carbonized. It’s evident that the PL intensity is dependent on the heat treatment of carbon fibers and decreases with increasing carbonization temperature. Such treatment could be assigned to the different degrees of crystal quality as well as defect centers of the carbon samples. Also, carbonyl functional group quantity affects the PL intensity which can be different for different carbonized fibers. The emission bands for carbonized fibers were slightly shifted due to the surface functional groups and different radiative recombination centers of electrons and holes trapped on the carbon surface. In carbon materials containing both sp^2^-and sp^3^ hybridization, the localized π and π* energy levels of the sp^2^ domains reside between the σ and σ* states of the sp^3^ matrix^48,49^. The optoelectronic properties are governed by the π states of the sp^2^ domains^50^. The PL from such materials arises due to radiative recombination of electron–hole pairs localized in sp^2^ domains. Photoemission can be manipulated by altering the fraction, size, and shape of sp^2^ clusters. It was demonstrated that PL energy varies inversely with the relative number of sp^2^ domains in disordered carbon materials^51^. Also it should be pointed out that emissive traps between π and π* of C–C, due to presence of various functional groups on the surface of the carbon fibers, could be another reason for the PL spectra of such fibers. Overall, the mechanism of the PL behavior of carbon fibers is very complicated due to the presence of different surface energy traps and layer plane length in carbon fibers.

**Figure 11:**
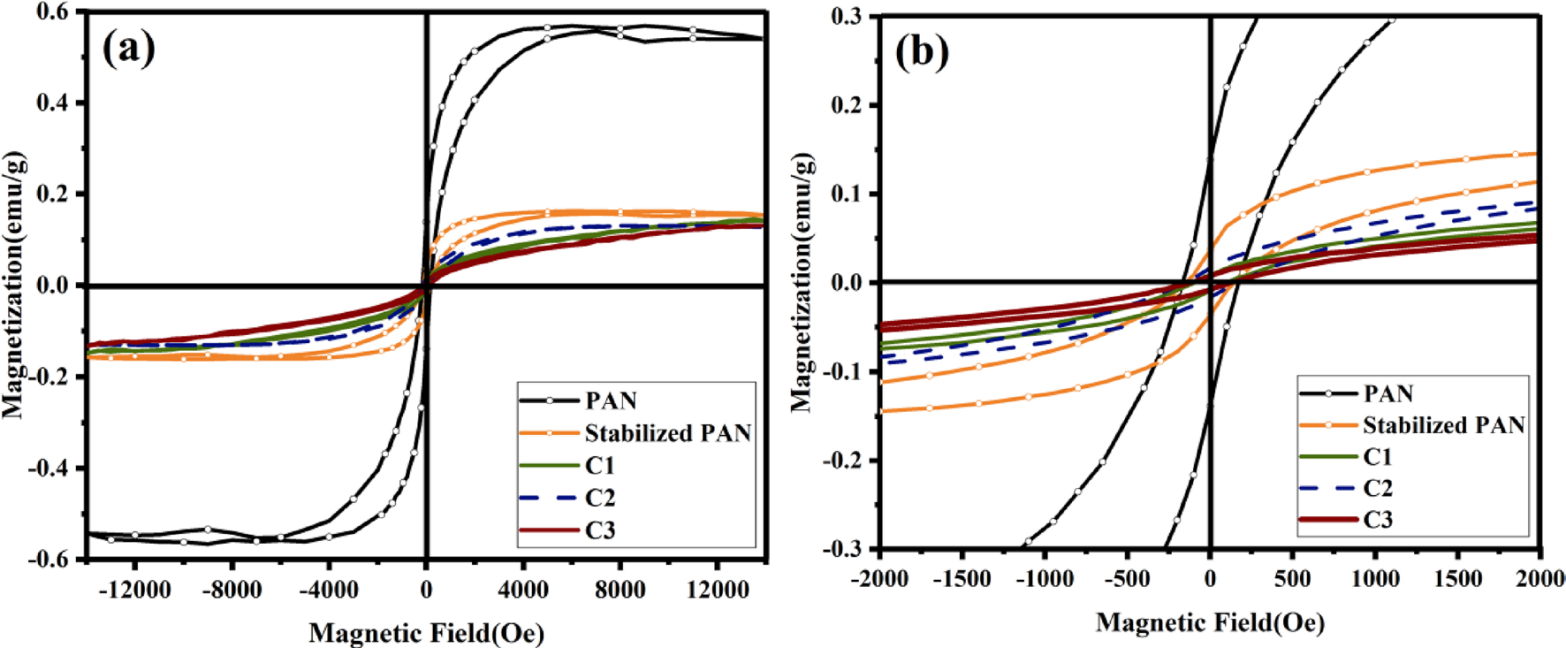
(**a**) Magnetization vs. magnetic field curve measured at room temperature of PAN-based carbon fibers, and (**b**) enlarged M–H curve of related samples.

**Figure 12:**
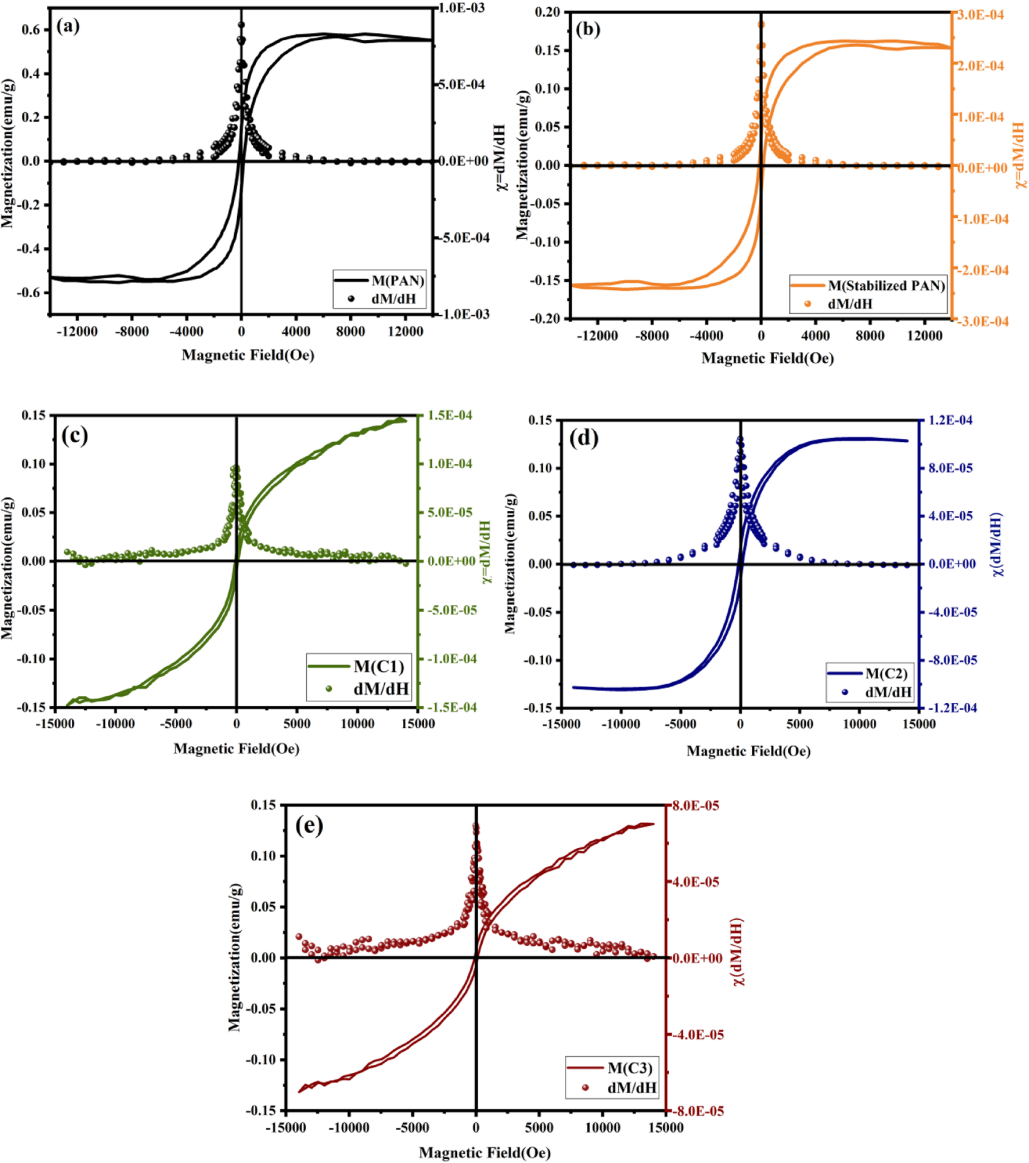
Magnetization vs. magnetic field curve, and the first derivative of M with respect to H for (**a**) PAN, (**b**) stabilized, (**c**) C1, (**d**) C2, (**e**) and C3 fibers.

**Figure 13:**
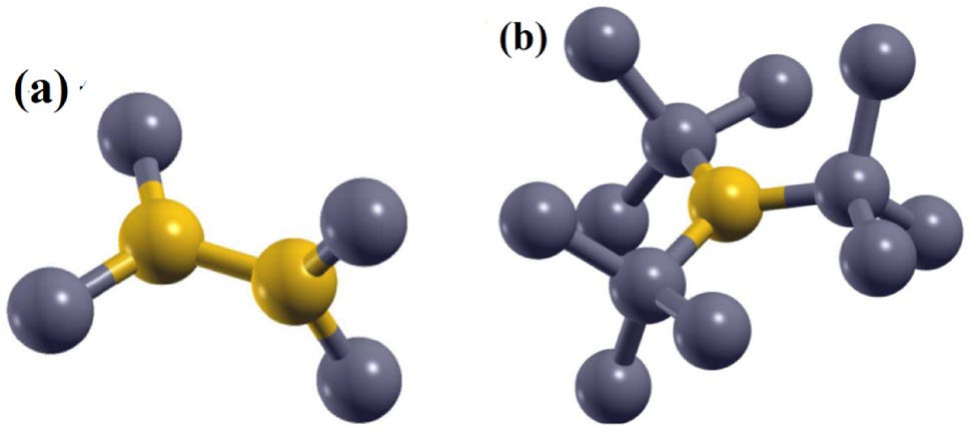
(**a**)Two threefold atoms bonded but their p orbitals are rotated relative to each other, and (**b**) a threefold carbon atom surrounded by fourfold atoms^55^.

The VSM analysis was used to investigate the magnetic properties of the PAN-based fibers. Figure 11 shows the variations of magnetization as a function of applied magnetic field and saturated hysteresis loop formation of different samples. The first derivative of magnetization (M) with respect to H for carbon fibers is plotted in Fig. 12a–e. Saturated hysteresis loop formation confirms the weak ferromagnetic nature of the as-electrospun, stabilized, and carbonized PAN fibers. The susceptibility (M/H) or slope of the curve decreases with increasing carbonization temperature. The remanent magnetization (M_r_), susceptibility (χ), and magnetic coercivity (H_C_) of carbon fibers are collected in Table 2. It’s evident from the magnetic characterization that as-spun PAN fiber shows a higher degree of magnetization, due to presence of nitrile groups, as dipole moments in nitrile groups of PAN cause its easy polarization in the magnetic field^52^. But as shown in the FT-IR spectra, nitrile groups disappear after stabilization and carbonization stages. Also it should be noted that magnetic parameters, H_c_ and M_r_, are influenced by the crystallite thickness (L_c_). In a comparison for the magnetic behavior of the carbonized samples (C1, C2, C3), one can see that the H_c_ and M_r_ reach to a maximum value, and then decrease with further increase in crystallite thickness for sample C3. Such magnetic behavior may be reflecting the transition from a single- to multi-domain structure inside the carbon fibers. The trends for carbon fibers is in consistent with previous experimental and theoretical study, indicating dependence of M_r_ and H_c_ values on the size and shape of particles^53,54^. The magnetic behavior of carbon materials containing sp^2^/sp^3^ hybridization is deeply related to the unpaired electrons in the threefold sp^2^-hybridized carbon. Two types of threefold atoms (sp^2^), including unpaired electrons, play a significant role in creating magnetic properties. As shown in (Fig. 13a), the first one is bonded whit their p orbitals rotated by 90˚ relatives to each other. Therefore, the relative rotation between two p electrons is necessary for showing the magnetic properties. The second one (Fig. 13b), is surrounded by three fourfold coordinated atoms creating an unpaired electron on the sp^2^ atom. The surrounding fourfold atoms do not have electrons to form additional bonds with the extra electron at threefold atoms. The ferromagnetic feature of the carbon materials is affected by the alternating sp^2^ and sp^3^-hybridized carbon atoms^55,56^.

**Table 2:** Obtained values for remanent magnetization, initial magnetic susceptibility and coercivity of the PAN-based carbon fibers.

Samples	M_r_ ${\times 10}^{-3}$(emu/g)	${\chi \times 10}^{-5}$(H = 0) (emu/gOe)	H_C_ (Oe)
PAN	138	88.9	175
Stabilized PAN	36	27.4	150
C1	9	6.5	100
C2	16	10.4	160
C3	7	8.8	120

The Electrical properties of PAN- based carbon fibers were determined by Hall Effect measurement. The electrical properties of PAN fiber were compared with carbon fiber, carbonized at 1000 °C “C1 sample”, as shown in the Table 3. The PAN fibers exhibit significant electrical resistivity and low electrical conductivity values of ${3.67\times 10}^{+6}$ Ω cm, $\mathrm{and}\; { 2.73\times 10}^{-7}$ S/cm respectively. While the C1 sample indicates the excellent electrical conductivity and lower electrical resistivity, as 133S/cm and ${7.52\times 10}^{-3}\Omega \; \mathrm{cm}$ in room-temperature, respectively. Sp^2^ hybridization for such sample was fevered to show good electrical conductivity. Also, this behavior could be due to σ electrons appear around an aromatic structure after heating of the graphitized-like crystallite. Then, holes form as a result of paired π and σ electrons that can hope between the graphitized-like crystallite by means of an electric field. Electrical behavior of carbon fibers is often different with the bulk one, due to difference in grain boundary and the degree of crystallographic order. The carrier in amorphous materials is transported by the thermally-assisted hopping of electrons between states localized near randomly distributed “traps”^57^. The electrical conductivity in carbon materials could be enhanced by a large number of conjugated double-bonds. Also, it should be noted the electrical conductivity is also very depended on the structural behavior.

**Table 3:** Hall-effect measurement results of PAN and carbon fibers carbonized at 1000 °C.

Sample	Resistivity (Ω cm)	Conductivity(s)
PAN	3.67E+6	2.73E−7
C1	7.52E−3	1.33E+02

## Conclusion

In summary, PAN-based carbon fibers were fabricated by using a simple and essential electrospinning technique, followed by stabilizing and carbonizing electrospun PAN fibers. The morphological characterization of the synthesized fibers shows the temperature sensitivity of carbonized fibers, as the fiber network shows few fiber breakage, due to the high-temperature activation that contributes to the removal of the volatile low molecular weight fractions. Also, the thermal residual stress may result in failure of the continues fiber morphology. The XRD patterns showed structural changes from linear structure into a graphite-like structure, as a removal of the non-carbon elements continues at carbonized stages. The explicit changes in the FTIR spectra confirm that different chemical reactions such as cyclization, cross-linking, as well as dehydrogenation take place in the chemical structure of PAN fibers. In a way that a fully carbonized of PAN, converted to the graphitic structure, in agreement with the XRD results. Absorption behavior of PAN-based carbon fibers obtained by DRS, show a well-defined *π*–π*/*σ*–π* and n–π* transitions related to formation of a conjugated electronic structure of fibers. PL spectra show slightly shifted of emission bands, which could be due to the surface functional groups and different radiative recombination centers on the carbon surface. The magnetic properties of the PAN- based carbon fibers show its dependency to the nitrile groups of PAN, indicating a weak ferromagnetic nature of the fibers. Also, magnetic properties of carbonized fibers confirm a transition from a single- to multi-domain structure and size effect. The Hall Effect measurements confirm that the electrical properties of PAN-based carbon fibers are affected by the sp^2^ hybridization, which fevered for showing a good electrical conductivity. Also, a higher value for electrical conductivity could be due to the generation of hole as a result of paired π and σ electrons that hope between the graphitized-like structure.

## Supplementary Information

Supplementary Figures.

## Data Availability

The authors declare that the data supporting the findings of this study are available within the article and its Supplementary Information file.
