# Supplementary material for: A comprehensive study on the effect of carbonization temperature on the physical and chemical properties of carbon fibers
Source: Sci Rep. 2022 Jun 23;12:10704. doi: 10.1038/s41598-022-15085-x (PMC9226016; doi:10.1038/s41598-022-15085-x)
Supplement: Supplementary file 1 — Supplementary Figures. [file 41598_2022_15085_MOESM1_ESM.docx]

SUPPLEMENTARY INFORMATION

**A comprehensive study on the effect of carbonization temperature on the physical and chemical properties of Carbon fibers**

Roya Shokrani Havigh, Hossein Mahmoudi Chenari*


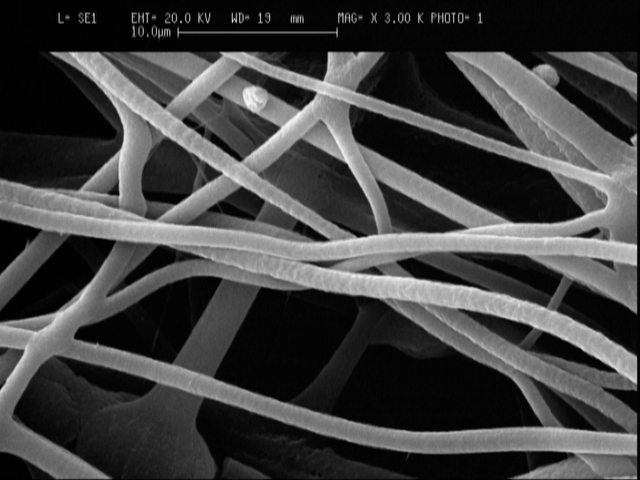


Supplementary Figure 1. SEM image of carbonized PAN fibers at 1000°C.


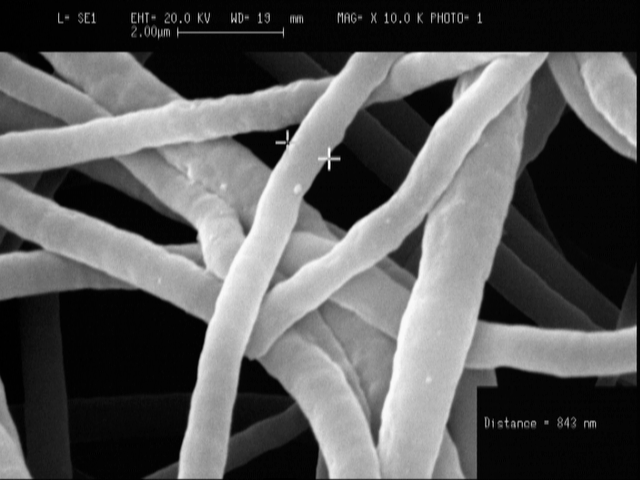


Supplementary Figure 2. SEM image of carbonized PAN fibers at 1400°C.


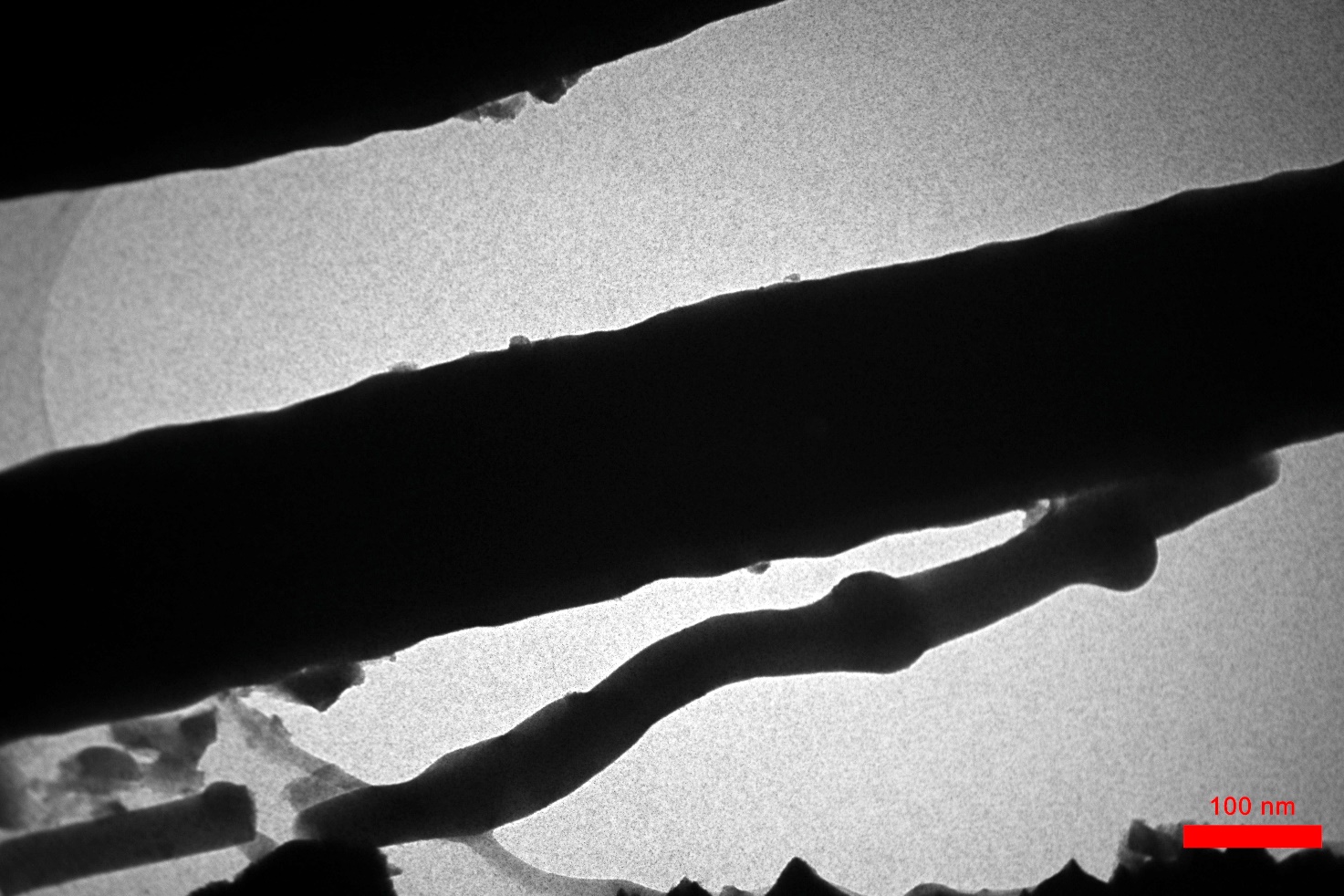


Supplementary Figure 3. TEM image of carbonized PAN fibers at 1000°C.


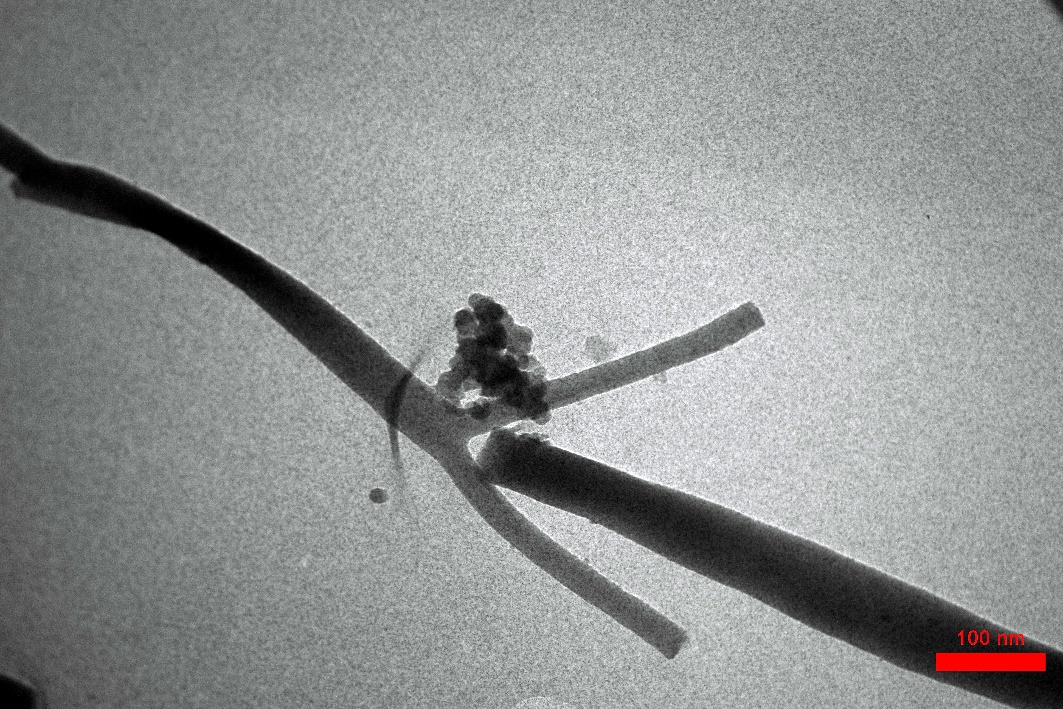


Supplementary Figure 4. TEM image of carbonized PAN fibers at 1400°C.


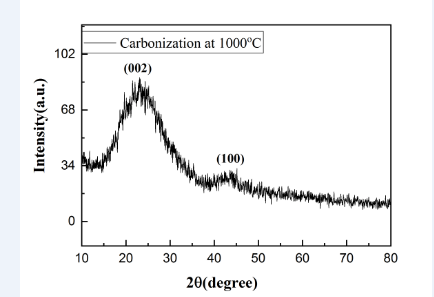


Supplementary Figure 5. XRD patterns of carbonized PAN fibers at 1000°C.


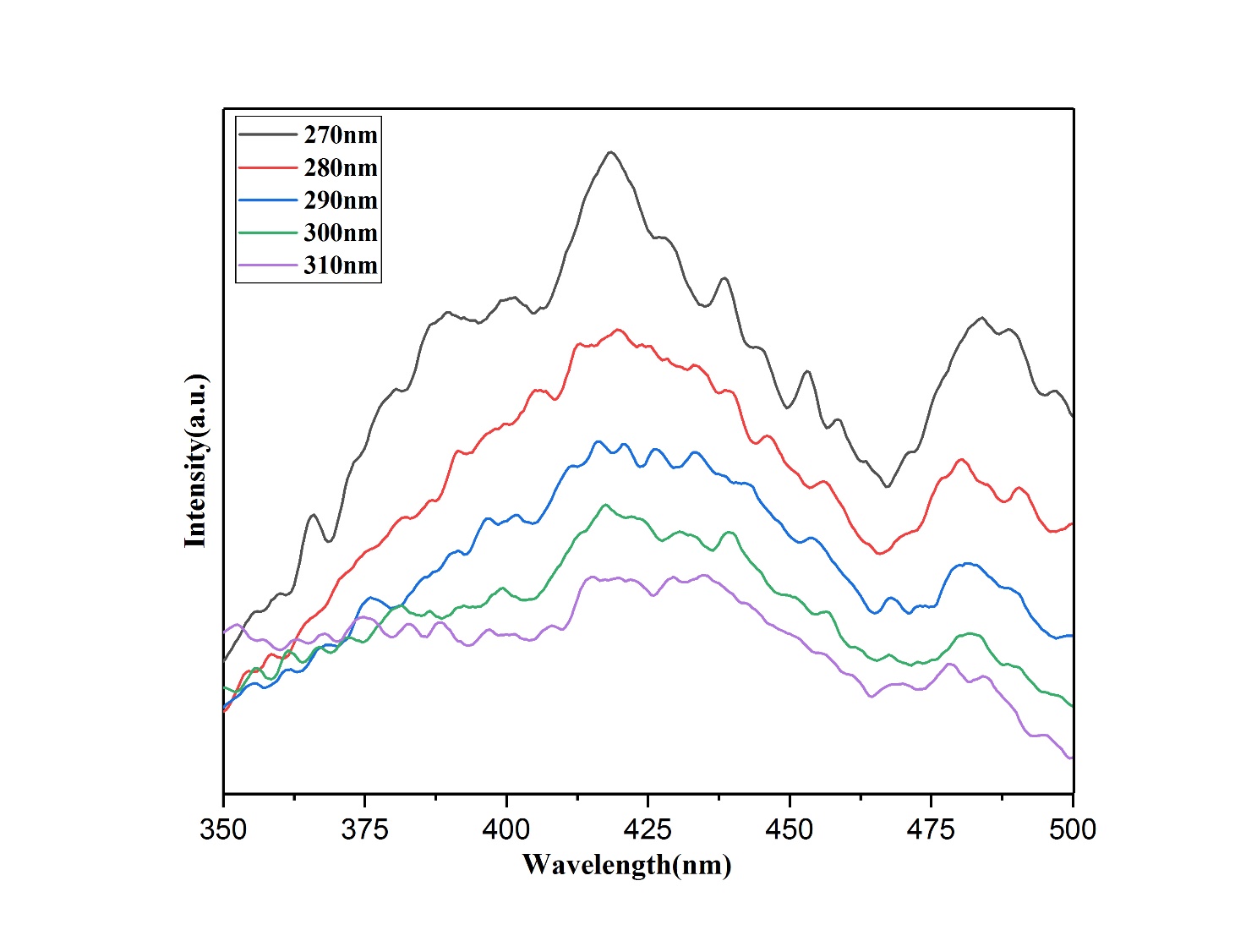


Supplementary Figure 6. the PL emission spectra of fibers carbonized at 1000°C at different wavelengths.
